# Supplementary material for: Multifunctional Portable System Based on Digital Images for In-Situ Detecting of Environmental and Food Samples
Source: Molecules. 2023 Mar 8;28(6):2465. doi: 10.3390/molecules28062465 (PMC10051621; doi:10.3390/molecules28062465)
Supplement: Supplementary file 1 [file molecules-28-02465-s001.zip › molecules-2217109-supplementary.pdf]

MULTIFUNCTIONAL PORTABLE SYSTEM BASED ON DIGITAL IMAGES FOR IN-SITU DETECTING OF ENVIROMENTAL AND FOOD SAMPLES

Diego Barzallo[1], Jorge Benavides[2], Víctor Cerdà[3]*, Edwin Palacio[1]*

[1]Environmental Analytical Chemistry Group, University of the Balearic Islands, Palma, Spain.

[2] Department of Electrical and Electronic Engineering. Universidad del Valle, Colombia.

[3]Sciware Systems, Bunyola, Spain.

*Corresponding authors:

victorcerdamartin@gmail.com

edwin.palacio@uib.es

Supplementary material

A)

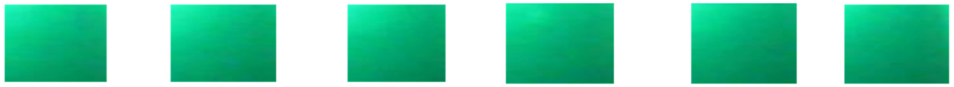

0.2 mg L^{-1} 0.3 mg L^{-1} 0.5 mg L^{-1} 0.7 mg L^{-1} 0.9 mg L^{-1} 1.0 mg L^{-1}

[NOR]	Red					Green					Blue				
mg L^{-1}	1	2	3	Average	SD	1	2	3	Average	SD	1	2	3	Average	SD
0.2	0.0578	0.0473	0.0332	0.0461	0.012	0.0107	0.0113	0.0102	0.0107	0.0005	0.0103	0.0119	0.0125	0.0116	0.0011
0.3	0.0668	0.0593	0.0788	0.0683	0.009	0.0161	0.0158	0.0171	0.0163	0.0007	0.0183	0.0163	0.0192	0.0180	0.0015
0.5	0.0472	0.0521	0.0647	0.0547	0.009	0.0215	0.0227	0.0237	0.0227	0.0011	0.0301	0.0295	0.0286	0.0267	0.0008
0.7	0.0521	0.0475	0.0501	0.0499	0.002	0.0309	0.0311	0.0323	0.0314	0.0008	0.0353	0.0363	0.0409	0.0358	0.0030
0.9	0.0293	0.0210	0.0255	0.0253	0.004	0.0386	0.0366	0.0382	0.0378	0.0011	0.0477	0.0491	0.0485	0.0471	0.0007
1	0.0521	0.0599	0.0570	0.0563	0.003	0.0464	0.0454	0.0457	0.0458	0.0005	0.0579	0.0574	0.0541	0.0549	0.0021

B)

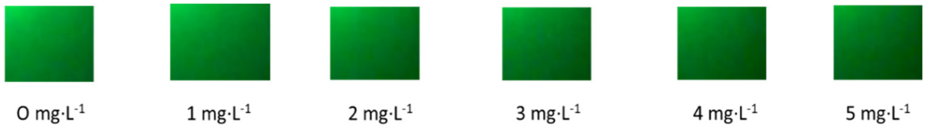

[Allura Red]	**Webcam System**														
	Red					Green					Blue				
$mg \cdot L^{-1}$	1	2	3	Avg	SD	1	2	3	Avg	SD	1	2	3	Avg	SD
0	0.00	0.00	0.00	0.000	0.000	0.00	0.00	0.00	0.000	0.000	0.00	0.00	0.00	0.000	0.000
1	0.006	0.006	0.008	0.007	0.001	0.031	0.030	0.030	0.030	0.000	0.047	0.044	0.046	0.046	0.001
2	0.091	0.073	0.082	0.082	0.009	0.062	0.060	0.060	0.061	0.001	0.097	0.094	0.092	0.094	0.002
3	0.061	0.045	0.025	0.044	0.018	0.074	0.071	0.071	0.072	0.002	0.135	0.129	0.132	0.132	0.003
4	0.176	0.161	0.180	0.172	0.010	0.115	0.111	0.111	0.112	0.002	0.181	0.176	0.176	0.178	0.003
5	0.214	0.178	0.187	0.193	0.019	0.139	0.137	0.137	0.138	0.001	0.205	0.206	0.201	0.204	0.002

Figure S1. Images obtained by performing absorbance measurements. A) Data obtained with norfloxacin standards using webcam system B) Data obtained with allura red standards using webcam system.

A)

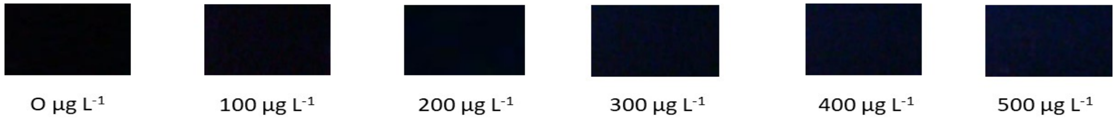

[Quinine]	**Webcam System**														
	Red					Green					Blue				
$\mu g \cdot L^{-1}$	1	2	3	Avg	SD	1	2	3	Avg	SD	1	2	3	Avg	SD
0	2.24	2.25	2.22	2.24	0.02	4.75	4.69	4.77	5.04	0.04	5.84	5.99	5.91	5.92	0.07
100	2.97	2.91	2.77	2.88	0.10	5.64	5.64	5.63	5.64	0.01	14.5	14.36	14.33	14.4	0.09
200	3.08	3.31	3.48	3.29	0.20	7.26	7.23	7.28	7.26	0.03	19.99	20.35	20.13	20.16	0.18
300	3.34	3.34	3.28	3.32	0.03	9.14	9.26	9.19	9.2	0.06	27.4	27.22	27.01	27.21	0.19
400	3.14	3.33	3.29	3.25	0.10	11.74	11.69	11.81	11.75	0.06	37.35	37.39	37.52	37.42	0.09
500	3.31	3.13.	3.22	3.27	0.06	13.96	14.11	14.11	14.06	0.09	46.49	46.32	46.86	46.56	0.28

B)

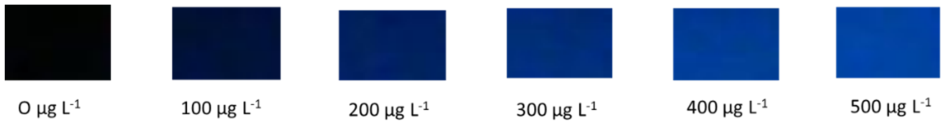

[Quinine]	**Webcam system**														
	Red					Green					Blue				
$\mu g \cdot L^{-1}$	1	2	3	Avg	SD	1	2	3	Avg	SD	1	2	3	Avg	SD
0	1.19	1.28	1.34	1.27	0.08	4.15	4.11	4.23	4.16	0.06	7.01	6.84	7	6.95	0.10
100	0.96	0.84	0.69	0.83	0.14	15.44	15.42	15.46	15.44	0.02	47.93	47.75	47.93	47.87	0.10
200	0.45	0.44	0.43	0.44	0.01	28.52	28.58	28.64	28.58	0.06	78.96	78.12	78.86	78.65	0.46
300	0.17	0.2	0.18	0.18	0.02	42.26	41.48	41.36	41.70	0.49	111.09	113.63	113.23	112.65	1.37
400	0.1	0.12	0.11	0.11	0.01	54.13	54.2	54.01	54.11	0.1	135.6	135.74	135.47	135.60	0.14
500	0.09	0.1	0.1	0.10	0.01	66.43	66.48	66.38	66.43	0.05	161.77	161.47	161.32	161.52	0.23

Figure S2. A) Images obtained of quinine standards using radiation with middle current intensity. B) Images obtained of quinine standards using radiation with high current intensity.

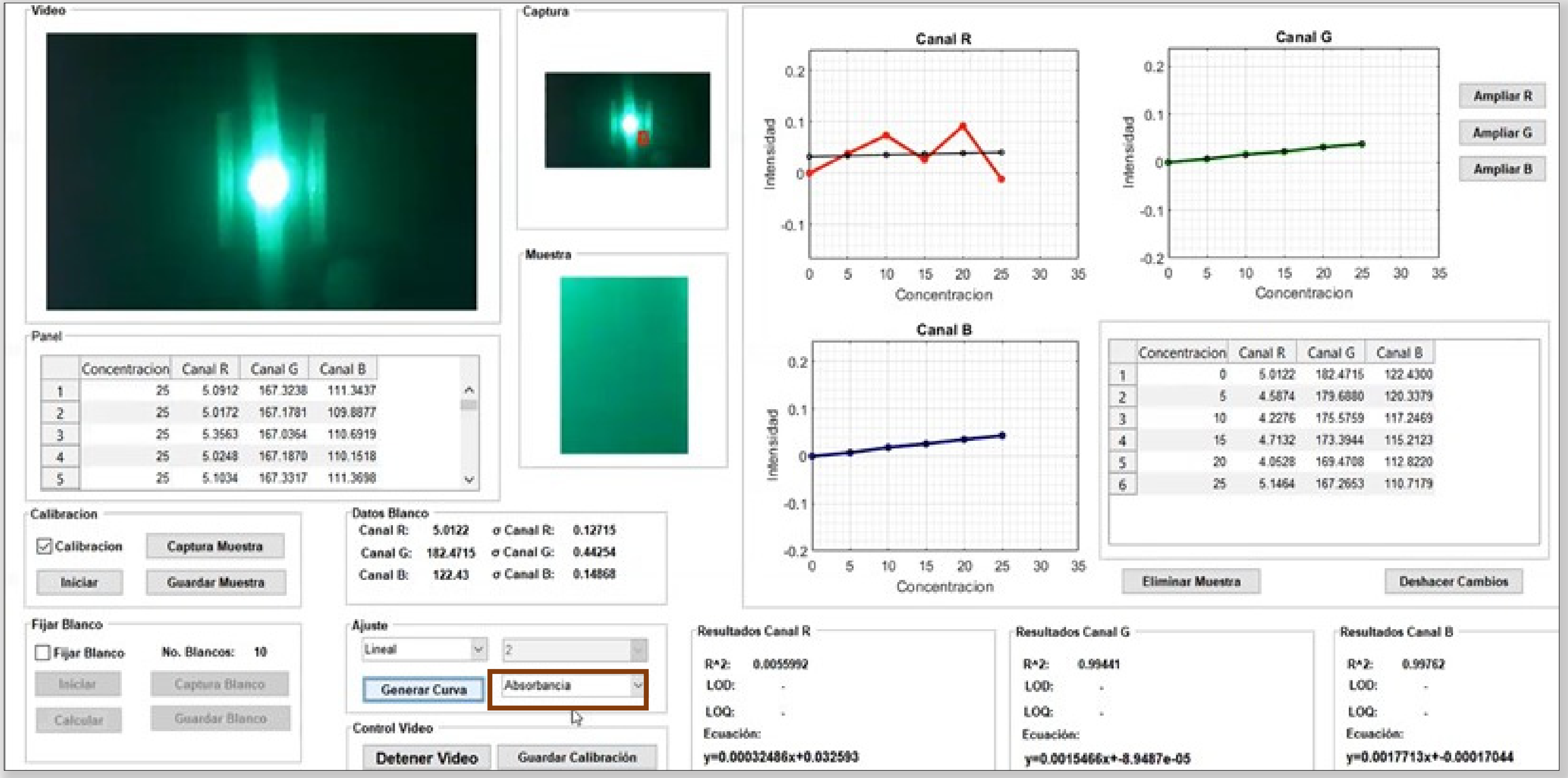

Figure S3. Execution of Matlab software in absorbance measurements. External calibration curve obtained from norfloxacin.

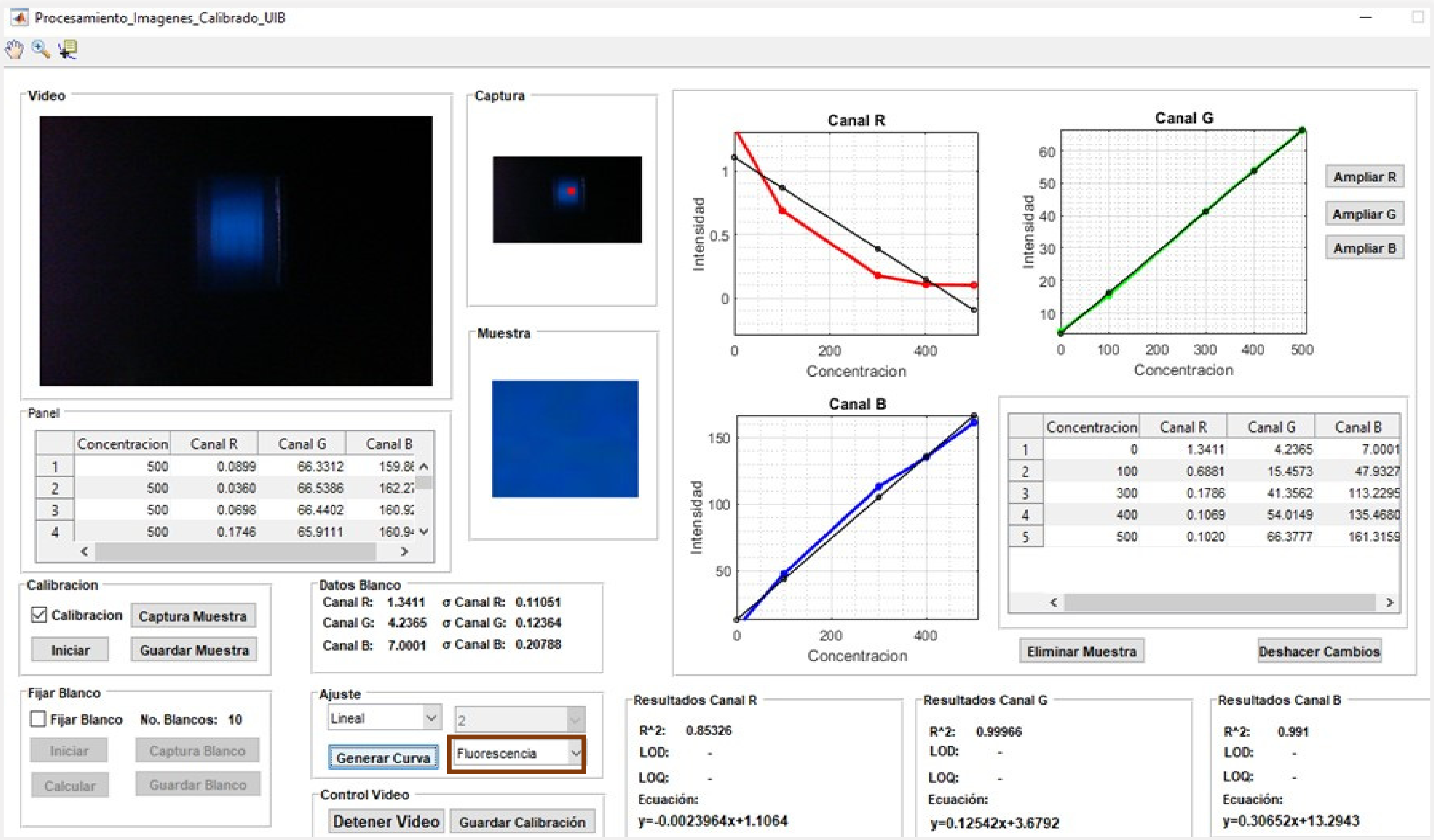

Figure S4. Execution of Matlab software in fluorimetric measurements. External calibration curve obtained from quinine sulfate

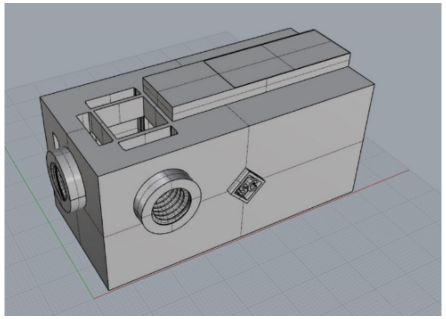

Figure S5. Design of 3D Holder using Rhinoceros program

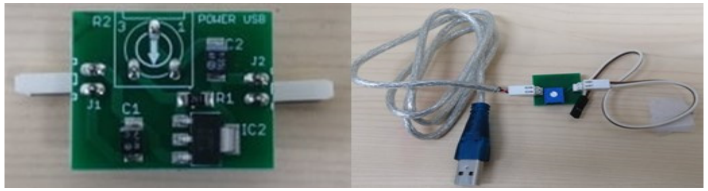

Figure S6. Electronic circuity used for experimentation

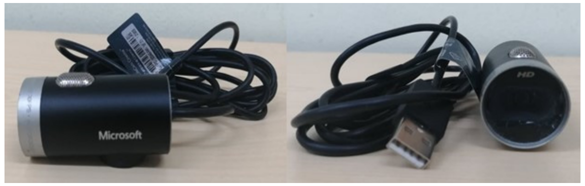

Figure S7. Photograph of the used webcam

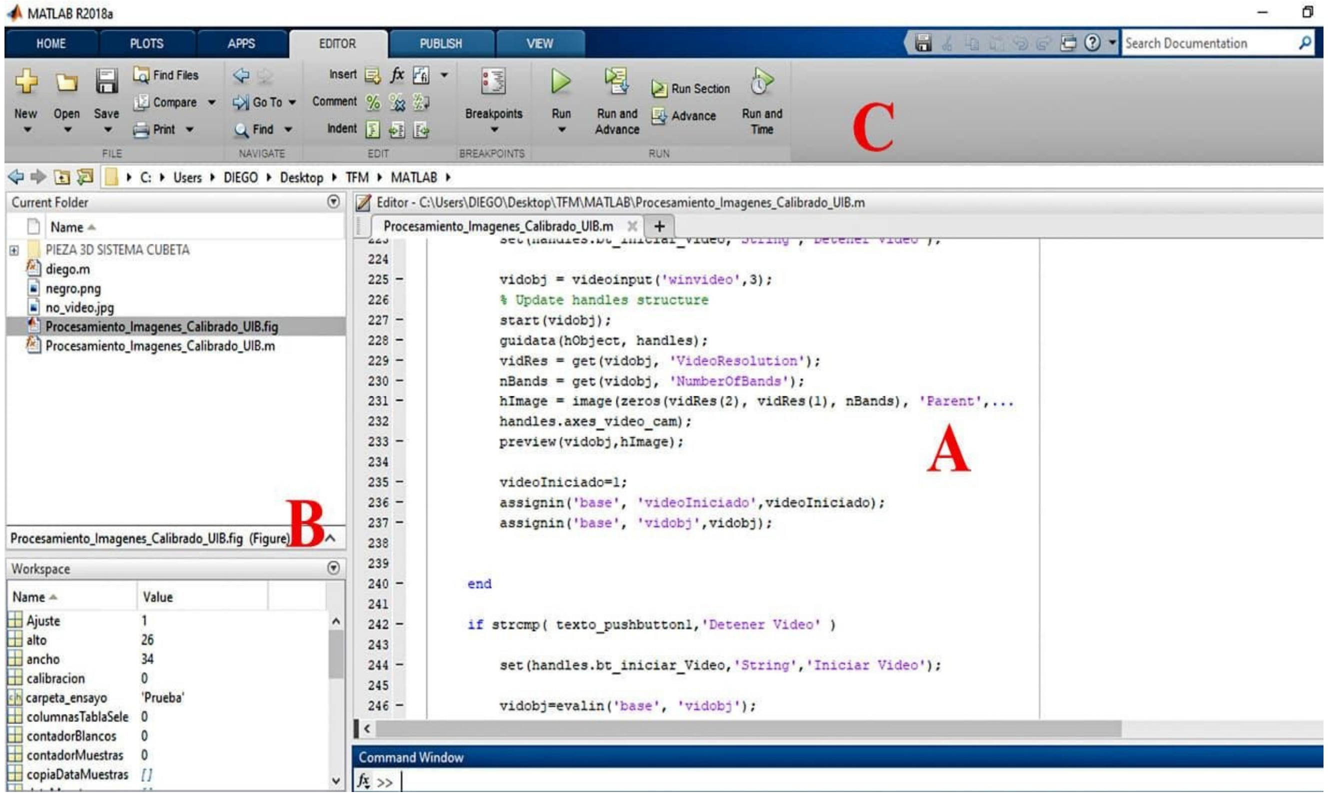

Figure S8. Programming language developed in Matlab. A) Command Window, B) Current Folder and Workspace and (C) Menu bar.
